# Development of the self-regulation assessment and content validation using cognitive interviews in a multicultural post-rehabilitation population

**DOI:** 10.3389/fresc.2023.1085658

**Published:** 2023-05-19

**Authors:** Tanja Mol, Eline Scholten, Coen van Bennekom, Marcel Post

**Affiliations:** ^1^ Center of Excellence for Rehabilitation Medicine, University Medical Center Utrecht, Utrecht, Netherlands; ^2^ Department of Rehabilitation Medicine, University Medical Center Groningen, Groningen, Netherlands; ^3^ Department of Research and Development, Rehabilitation Centre Heliomare, Wijk aan Zee, Netherlands; ^4^ Department of Public and Occupational Health, VU Medical Center, Amsterdam, Netherlands

**Keywords:** rehabilitation, content validity, self-regulation, multi-diagnostic, measurement

## Abstract

**Aim:**

Self-regulation is one of the main goals of medical rehabilitation. Four themes of self-regulation were identified by former patients and rehabilitation physicians in a previous study. Based on these themes, a measure for self-regulation, the self-regulation assessment (SeRA), was developed. This study aimed to establish the content validity of the SeRA in a multicultural and multi-diagnostic post-rehabilitation population.

**Methods:**

The Consensus-based Standards for the selection of health Measurement Instruments (COSMIN) methodology was applied. First, cognitive interviews were held with eight former rehabilitation patients. Feedback was obtained on relevance, comprehensibility, and comprehensiveness of the items. Items with problems were revised. Then, a second series of cognitive interviews was held with 16 former rehabilitation patients with non-Western migration backgrounds. Again, feedback was obtained on relevance, comprehensibility, and comprehensiveness of the items.

**Results:**

The first series of cognitive interviews revealed good comprehensiveness, and also comprehensibility or relevance problems with 12 of the 25 items. These items were revised or deleted. Two missing concepts were identified and these were added. There was no need to revise the items based on the results of the second series of cognitive interviews.

**Conclusion:**

The final version of the SeRA demonstrated content validity for the studied population. The measure is ready for psychometric analyses in subsequent validation studies.

## Introduction

Persons living with a chronic health condition have to adapt to physical as well as psychological changes in their bodies and their lives. Medical rehabilitation treatment helps by preventing, reducing, and eliminating limitations caused by this health condition (1, 2). The overall aim of rehabilitation is to improve a person's self-regulation, societal participation, and health-related quality of life (HRQoL) (3). Rehabilitation treatment comprises a learning process, including educational and self-regulation interventions in addition to training of physical and cognitive functioning (4).

Self-regulation can be defined as a continuously active process of managing and changing the self (5). Self-regulation beliefs determine how people think and behave. However, self-regulation is a complex concept with diverging definitions and meanings for different target populations (6–9). In a previous focus group study among former rehabilitation patients and a Delphi study among rehabilitation physicians, aspects of self-regulation important in the context of rehabilitation were explored (10, 11). A comprehensive model of self-regulation in a rehabilitation context was developed, which comprises four important themes. Two of these themes are conditional for self-regulation: (1) to have self-insight into one's condition and abilities (insight in impairments; in the consequences of these impairments; and in abilities); (2) to know how to cope with the consequences of the condition (being able to communicate limitations; and to have trust in body and functioning); (3) concerning about how to apply self-regulation in one's own life (making use of abilities and optimize functioning) (10); (4) to focus on the organization of help (including asking for help and directing help) (11).

In general, measurement of patient-reported outcomes can help improve quality of care and identify best practices, to monitor patients’ progress, and is important for clinical research (12, 13). Measurement of self-regulation as a rehabilitation outcome is sparse, and it is also unclear which measure of self-regulation is to be preferred in the context of rehabilitation outcomes measurement. A systematic review to identify measures of self-regulation used in rehabilitation populations showed that none of the existing measures covered all of the four themes of self-regulation and was applicable in multiple diagnostic groups (14). Therefore, a new measure was developed based on the four themes, the self-regulation assessment (SeRA). First, based on the content of the conceptual framework and measures identified in the systematic review, a list of 65 concept items was established (14). An expert group of patient representatives, rehabilitation professionals, rehabilitation managers, and researchers (*n* = 10) discussed the 65 concept items and agreed upon a selection of 25 items. This 25-item list was used as the first draft version of the SeRA. Based on feedback from the earlier conducted Delphi study, a five-point Likert scale, from totally disagree up to totally agree, was chosen as the most appropriate response scale of all items.

In the development of a new measure, it is important to make sure that the right items are selected and that the target respondents understand and interpret the items correctly. Content validity is defined as “the degree to which the content of an instrument is an adequate reflection of the construct to be measured” (p. 4) (12) and can be operationalized as the relevance, comprehensibility, and comprehensiveness of a measure for the target population. It is often considered the most important measurement property and therefore an important step in the development of a measure (12).

This study aimed to determine the content validity of the SeRA by assessment of the relevance, comprehensibility, and comprehensiveness of the items, following the Consensus-based Standards for the selection of health Measurement Instruments (COSMIN) methodology for assessing the content validity of PROMs (15). The SeRA would desirably be used throughout the whole adult rehabilitation population, irrespective of diagnosis, sociodemographic characteristics, and cultural background. Persons from non-Western migration backgrounds represented 14% of the Dutch population in 2021, and they may have different needs and expectations of rehabilitation healthcare (16, 17). This group is commonly underrepresented in research, also in the first series of the cognitive interviews of this study, and therefore tailored recruitment strategies to enhance their participation have been recommended (18, 19).Therefore, the research questions (RQ) to be answered in this study are:

**RQ1**. How do former rehabilitation patients rate the relevance, comprehensibility and comprehensiveness of the items of the SeRA?

**RQ2.** How do former rehabilitation patients from non-Western migration backgrounds rate the relevance, comprehensibility and comprehensiveness of the items of the SeRA?

## Material and methods

This qualitative study is part of a larger study named “Measurement of Outcomes of Rehabilitation in the Netherlands” (MUREVAN). The “consolidated criteria for reporting qualitative research” (COREQ) were applied in the description of this qualitative research (20).

### Design

Cognitive interviews were conducted to identify respondents’ interpretations of the items of the SeRA. This technique is advised to test and refine measures on content validity and interpretability (21).

## Cognitive interviews (research question 1, RQ1)

### Selection of respondents and procedure (RQ1)

Purposive sampling was used. Individuals with a chronic physical health condition, i.e., spinal cord injury or neuromuscular disease, who had undergone a medical rehabilitation treatment at some moment in their life were included. In addition, they had to be at least 18 years old at inclusion in this study. Individuals with insufficient knowledge of the Dutch language were excluded. Variation with respect to diagnosis, age, gender, level of education, and type of rehabilitation trajectory (inpatient or outpatient) was aimed for.

Recruitment was done via the personal network of the interviewer and among former patients of the De Hoogstraat Rehabilitation center. Possible respondents were invited by email. Eight face-to-face cognitive interviews were held during December 2019 and January 2020. Interviews were held at the De Hoogstraat Rehabilitation center or in the respondents' homes, as they wished. Informed consent was obtained. Respondents were explained that the aim of the interview was not to test their self-regulation abilities, rather to understand their thoughts about the SeRA.

The preliminary 25-item version of the SeRA was used in the first series of cognitive interviews. Respondents were asked to think aloud and explain all of their thoughts when answering the items. Probing was used when respondents did not spontaneously express their thoughts on the items. Reflective questions on the SeRA in general were asked directly after the cognitive interview, e.g., whether the items fully reflect the concept, what their thoughts were concerning the items in general, reflection on the instructions and the response options, and if they missed items. Interviews were held in the Dutch language and were audiotaped. Interviews lasted between 40 and 90 min. Prior to the interviews, a few test and feedback interviews were conducted to ensure qualification of the interviewer. All eight interviews were held by the same interviewer (KH), and during the first three interviews, her supervisor (TM) was the second interviewer.

### Data management and content analyses (RQ1)

All audio recordings were transcribed verbatim and anonymized. Data analyses were performed by using MaxQDA software (Verbi Gmbh MaxQDA 2018.2). Thematic analysis and open coding were used for content analysis. Cognitive interviews were initially analyzed by the interviewer, and next by the first author of this manuscript. The coding system of the analyses was based on three categories in which 10 criteria for good content validity are divided: (a) comprehensibility, (b) comprehensiveness, and (c) relevance (12). Comprehensibility covers four criteria: instructions understood as intended, items and response options understood as intended, items worded appropriately, and match between questions and response options. Comprehensiveness refers to the criterion whether no key concepts are missing. Finally, relevance contains five criteria: all items relevant for the construct, for the target population, and for the context of use; are response options appropriate; and if the recall period is appropriate (12). The coding system of Willis is recommended in the literature to analyses questionnaire problems as it provides detailed directions for revisions (22). This coding system was used to categorize problems or difficulties with the items. The codes that were used are: clarity (problems with the meaning of an item); knowledge (to not know or have trouble remembering information); assumptions (underlying logic not or incorrectly understood); response categories; and sensitivity (in wording or bias) (22). All findings were discussed within the project team (all authors of this publication). All authors involved in the content analyses were, or were supervised by, experienced qualitative researchers and were from different backgrounds such as psychology, health sciences, and rehabilitation medicine.

## Cognitive interviews (research question 2, RQ2)

### Selection of respondents and procedure (RQ2)

Also in the second phase, purposive sampling was used. Respondents were included if they were from a non-Western migration background, defined as that they, or at least one of their parents, were born in a non-Western country (23). Other inclusion criteria were that they had undergone a medical rehabilitation treatment at some moment in their life, were at least 18 years old at the inclusion in this study, and had sufficient knowledge of the Dutch language. Variation with respect to diagnosis, age, gender, educational background, and type of rehabilitation trajectory was aimed for.

Recruitment of respondents was done via patient organizations, social and cultural foundations for people with an immigrant background, social media (LinkedIn and Facebook), and personal contacts. Contact prior to the interviews was made to provide more information and assess their eligibility. Cognitive interviews were held in April and May 2020, and in April and May 2021. After analysis of the first eight interviews, we believed saturation was not yet reached. Therefore, eight additional interviews were conducted. A total of 16 interviews were held face-to-face or via video calling, due to COVID-19 restrictions. After an introduction, consent was obtained.

Each interview started with general questions about their rehabilitation experience and knowledge of self-regulation. Next, respondents were explained that the aim of the interview was not to test their abilities, rather to understand their thoughts about the SeRA. The revised version of the SeRA which contains 22 items was used. Similar interview techniques for cognitive interviews were used as for answering RQ1. Interviews lasted between 30 and 80 min. The 16 interviews were held by three different interviewers, all supervised by TM. Prior to the interviews, a few test and feedback interviews were conducted.

### Data management and content analysis (RQ2)

Similar techniques for transcribing and analyzing the interviews were used as for RQ1.

Cognitive interviews were initially coded by the interviewer. Ten of the interviews were coded by a second interviewer. TM conducted analyses of all 16 interviews for the current publication as well. The coding system of the analyses was similar as for RQ1 based on three categories: (a) comprehensibility, (b) comprehensiveness, and (c) relevance. If items were interpreted differently than intended, the reason for this discrepancy (for example, the cultural background and religion) was used in the coding system. All findings were discussed within the project team (all authors of this publication).

## Data quality assurance

The criteria used to determine the rigor of the study included the credibility, transferability, dependability, and conformability of the data (24). To ensure the data credibility, each transcript was independently analyzed by at least two researchers. Furthermore, the coding systems were discussed with all authors of this publication until agreement in understanding was reached. Also, quotes of respondents were provided. Transferability was ensured by providing description of the study setting, including all main diagnostic groups and persons from different ages and migration backgrounds. To ensure data dependability, accurate documentation was provided of the research methods, of all changes and revisions, and of all the results. To maintain conformability, probes were used to obtain detailed information. The written transcripts of the audio recordings were safely archived. Audio files were deleted for privacy reasons.

### Statement of ethics

The study protocol was reviewed by the Ethics Committee of the University Medical Centre of Groningen, and it was declared that this study did not need approval according to the Dutch law (registration number 201800582).

## Results

### Respondents (RQ1)

Eight respondents were included. Half of the respondents were male. The diagnostic group of brain injury was the largest group with four respondents. All patient characteristics can be found in Table 1.

**Table 1:** Participant characteristics.

	RQ1^a^ (*n* = 8)		RQ2^a^ (*n* = 16)	
Patient characteristics	*N*	% or mean (range)	*N*	% or mean (range)
Male	4	50	4	25
Age	7 (1 missing)	53 (32–66)	16	44.1 (24–65)
Patient admission: inpatient	6	75	4	25
Diagnoses				
Spinal cord injury	2	25	1	6.3
Chronic pain disorder	0	0	1	6.3
Musculoskeletal disorder (including amputation)	1	12.5	6	37.5
Neurological (including neuromuscular)	0	0	1	6.2
Brain injury	4	50	4	25
Other (including oncology and organ diseases)	1	12.5	3	18.7
Time since (last) rehabilitation treatment (years)	8	11.3 (0–36)	16	4.5 (0–12)
Migration background				
Native Dutch	8	100	0	0
Middle-Eastern	0	0	4	25
African	0	0	5	32.5
South-American	0	0	7	42.5

^a^ Research question.

### Content validity (RQ1)

#### Comprehensibility

Sixteen out of the 25 items did not raise any comments regarding comprehensibility, and the answers given by respondents matched the intention of the questions. For the other nine items, most problems concerned understanding issues due to complex formulations or difficult wording. Table 2 provides an overview of these nine items and the problems, including quotes.

**Table 2:** Content validity of the SeRA items RQ1^a^.

Item	Comprehensibility^b^	Relevance	No. of respondents	Example quote
I get my condition.	Clarity: nuance in formulation of the word choices.		3	Yes, I do not understand all of it, I do not know how it exactly works […] I understand I have a stroke (Male, brain injury, 63 years).
I got to know my body again.	Sensitively: the word “again” is not the right word.		5	Well, I would not say again, it is more like a gradual process, so that makes it more difficult (Male, neuromuscular disorder, 32 years).
I understand the impact of my condition.	Clarity: not specific.		2	It does not just impact my muscles… it also impacts my energy level, and I get cold more easy. So which aspect do you mean with this question? (Female, musculoskeletal disorder, 58 years).
I know what I am able of.	Clarity: not specific	Construct: overlapping with another item.	4	I believe it is a weird question, I mean… Of course, that is why we have brains (Female, oncology, 36 years).
I can and dare to indicate my limits.	Assumptions: double-barreled. Clarity: not specific.		6	This is ambivalent. Can and dare are definitely two different meanings (Male, brain injury, 60 years).
I know when I need help.		Construct: overlapping with another item.	5	I know when I need help, I determine when help is needed. Is that not the same question? (Female, brain injury, 34 years).
I determine when help is needed.		Construct: overlapping with another item.	5	—
I know where I can find help.	Clarity: not specific.		1	I am not sure how to interpret this, help could direct to professional help or to medical devices (Female, brain injury, 34 years).
I dare to trust myself.	Clarity: nuance in formulation of the word choices/unclear.	Construct: nothing to do with “daring.”	6	That is a difficult one, I always dared to trust on myself, but I was not right (Male, brain injury, 60 years).
I make the right choices.	Clarity: nuance in formulation of the word choices/unclear.		7	I make the right choices, I don’t know, what do they actually mean? (Female, oncology, 36 years).
I do the things I want to do.	Clarity: no reference.		2	Yes, so is this long term or short term? That could be really different (Male, brain injury, 60 years).
I determine which help is needed.		Construct: overlapping with another item.	1	I think I have had this question already? Or am I crazy? Yes, here number … (Male, brain injury, 63 years).

^a^ Research question.

^b^ Problems are specified based on the problems identified by Willis (22).

The instructions of the SeRA were understood as intended by each respondent. The response of one was:

“Yes, what do I think of this? It describes the whole concept briefly, and also what you expect from the reader” (Male, neuromuscular disorder, 32 years).

#### Comprehensiveness

Half of the respondents believed this measure was comprehensive and no concepts were missing. Four respondents marked two missing concepts. First, communication to others about the consequences of the condition was marked by two respondents. They explained that not all impairments are visible and that is important to share their needs. One said:

“If I am able to explain to others how others can treat me best. I believe that is super relevant too” (Female, brain injury, 34 years).

Second, to gain insight into emotional consequences and insecurities was marked as missing. Respondents explained that they had experienced many changes in their lives due to their condition, and that this all came with a lot of emotions. The comment of one was:

“I miss some questions on how people, the patients, cope with their condition and impairments in their heads, emotionally. That is for me a major concern at this moment. That I do have way more emotions than I used to have before” (Male, brain injury, 63 years).

#### Relevance

Respondents underscored the relevance of the items for a rehabilitation population. They particularly emphasized the items on gaining insights and understanding, and items on determination of own boundaries or possibilities. The respondents marked them as very relevant for medical rehabilitation treatment, and also for the construct of self-regulation. Comments from two respondents were:

“I believe that one feels like being able to self-regulate. Definitely. It makes you aware […] So also from a rehabilitation point of view is this well done. Definitely” (Female, brain injury, 34 years).

“I mean, if you do not understand your own condition, you will never be able to self-regulate” (Female, brain injury, age unknown).

Respondents also expressed that all items were relevant. An example quote is:

“If you do not have this, insight in own impairments, you will never be able to create insight into the consequences of your condition and possibilities. So I can say each question is conditional for the next step” (Male, brain injury, 60 years).

Also, items were marked as relevant for the rehabilitation population. Two respondents commented as follows:

“It makes you aware that it helps you when you understand your condition, but also that you realise what is still possible, etc. And that it is emphasised that you know where you can get help and from who. Those kinds of details” (Female, brain injury, 34 years).

“Well, I did not see any question that made me think ‘how can you ask this’?” (Female, brain injury, age unknown).

Four items were marked as overlapping with other items, because they were similar to other items, or due to their wording. An overview of these items can be found in Table 2.

Concerning the response options, a few remarks were made that these were not appropriate for each item. Two respondents indicated that answering the questions is not a matter of indicating agree or not agree and missed the option to clarify their answers. Finally, respondents seemed not to have any difficulties regarding the recall period. None of the respondents had problems to remember anything needed to answer the items.

#### Revisions after the first series

Based on the identified issues with comprehensibility and relevance, adaptations in the SeRA were made. Four items were omitted and two items were added. Adaptations are displayed in Table 3. One adaptation to the SeRA was conducted without the remarks of the respondents. The item “I ask for help when I think I need it” was specified into “I ask *others* for help when I think I need it”.

**Table 3:** Adaptations made in the SeRA based on the cognitive interviews (RQ1^a^).

Item with problem	Solution	New item
I get my condition.	More sensitive wording.	I understand my condition.
I got to know my body again.	Delete item.	—
I understand the impact of my condition.	Split into two items which are more context specific.	I understand the *physical* impact of my condition. I understand the *emotional* impact of my condition.
I know what I am able of.	Delete item.	—
I can and dare to indicate my limits.	Specify.	I dare to indicate my limits *to others*.
I know when I need help.	Specify.	I know when I need help *from others*.
I ask for help when I think I need it.	Specify.	I ask *others* for help when I think I need it.
I know where I can find help.	Specify.	I know *who to ask* for help.
I dare to trust myself.	Delete item	—
I make the right choices.	More sensitive wording.	I *trust* the choices I make.
I do the things I want to do.	More specific.	*If I want something, I know how to go about it.*
I determine when help is needed.	Delete item	—
I determine which help is needed.	Delete item	—
—	Add item.	I can explain the consequences of my condition clearly to others.

^a^ Research question.

### Respondents (RQ2)

Sixteen respondents were included in the study. Of the respondents, 75% (*n* = 12) was female. Respondents had a Middle-Eastern (*n* = 4), African (*n* = 5), or South-American (*n* = 7) migration background. All characteristics can be found in Table 1.

### Content validity (RQ2)

#### Comprehensibility

Respondents generally understood the items as intended. Interpretations of the items as expressed by the respondents matched with the intentions and responses matched with the question, as is illustrated by the following quotes:

“(*I know how to deal with my impairments)*. I know that for sure. If I am tired, than I stop what I am doing and I will take some rest. It does not matter if it is a party or something else, I will just go home” (Female, brain injury, 64 years, South-America).

“(*I dare to indicate by boundaries to others*). Yes, definitely, if I say I do not feel comfortable with, or I do not dare something. I dare to indicate this to others” (Female, amputation arm, 32 years, Middle-East).

At the end of the cognitive interview, respondents were asked whether they understood everything and whether they would change, add, or delete something. Respondents emphasized they understood most of the items, except the items containing the Dutch word for capabilities (“mogelijkheden”). This word was unclear for five respondents. However, after probing what respondents believed it would mean, they expressed the meaning as it was intended. As one respondent commented:

“Maybe the things I can do? I would interpret it in that way” (Female, brain injury, 33 years, Middle-East).

Furthermore, two respondents marked a lack of contextual information in several items. For them, it was unclear if the intention was to ask “in general” or “focused on the condition or impairment.” For example, regarding the item “I am aware of my capabilities,” it was unclear for these respondents whether the item referred to personal capabilities in life or to treatment options. A few respondents referred to their life in general and not just to their condition or impairment. A few example quotes are given:

“(*I trust on my own thoughts*). Definitely, I have developed this. Well, I have a 7th sense. I can sometimes read other people's mind. If you have passed away several times, you will get this sense from God” (Male, spinal cord injury, 60 years, Middle-East).

“(*I know what my options are*). Neutral answer, you never know when something happens to you. You are not in control” 9Female, COVID-19, 24 years, Middle-East).

One respondent did relate her answers to one specific consequence of her condition. She had epilepsy and answered the questions for the situations of having a seizure.

“(*I know when to ask for help*). The moment of the seizure not. I did not know when to ask for help. A seizure could come at any time. So when I answer this question related to the seizures, I would answer with ‘disagree’. But when it has nothing to do with my seizures, then I will just ask persons in my surroundings. It is more … I would say it is situational dependent” (Female, neurological disorder, 53 years, Middle-East).

#### Comprehensiveness

Respondents were positive about the items and the measure as a whole. In general, they emphasized that no items or concepts were missing. The following quote is an example:

“I think this fits with the concept of self-regulation. There are many different kind of questions in the measure, which is good. It is set up broad” (Female, neurological disorder, 53 years, Middle-East).

However, one respondent advised to add an item on the difference in self-regulation prior to rehabilitation compared to after the rehabilitation treatment.

#### Relevance

None of the respondents expressed doubts about the relevance for any of the items. Respondents emphasized relevance of the SeRA for rehabilitation treatment. The following are three examples of their comments:

“I believe it were good questions, it reflects your own process” (Female, COVID-19, 24 years, Middle-East).

“I believe this is something which has been central throughout my own rehabilitation treatment” (Female, musculoskeletal disorder, 26 years, Africa).

“ … from rehabilitation on, to get back your own life. With your own awareness, your own boundaries and feelings” (Female, chronic pain disorder, 35 years, Middle-East).

All items were highlighted as important. Items on understanding your condition, knowing how to deal with consequences, determining what you want, having trust in oneself, access to help, and make your own choices were emphasized by multiple respondents as very important for self-regulation. Quote example:

*Do you think this is self-regulation*? “Yes, I believe so. There were many topics throughout the measure, but it makes it clear if someone is able to self-regulate their life” (Female, musculoskeletal disorder, 27 years, Africa).

Finally, answers provided suggested that the recall period was appropriate, and nobody had any difficulties in remembering any of the answers. Response options were judged as good, and no suggestions were made to change these. Two quote examples:

“Response options were clear and brief” (Female, neurological disorder, 53 years, Middle-East).

“Questions were brief, which made them very clear. And the five response options were easy to understand” (Female, brain injury, 53 years, South-America).

#### Revisions after the second series

None of the items was revised based on the second series of cognitive interviews. Instructions of the SeRA were revised. A sentence on the perspective on how to answer the items was added.

## Discussion

The first series of cognitive interviews revealed good comprehensiveness, and also comprehensibility or relevance problems with 12 of the 25 items of the preliminary SeRA. These items were revised or deleted. Two missing concepts were identified, and items to measure these concepts were added. The second series of cognitive interviews, held with former rehabilitation patients with a non-Western migration background, confirmed the relevance, comprehensibility, and comprehensiveness of the final version of the SeRA items. Instructions of the SeRA were revised after the second series of interviews to clarify the perspective on how to answer the items.

Psychosocial concepts such as self-regulation can be complex to operationalize in a measure (25). The SeRA was created as a patient-reported outcome measure with the assumption that patients can define best what they need, in this case with respect to assessing and maximizing self-regulation during rehabilitation (12). The cognitive interviews supported content validity. However, the results of the interviews also revealed some issues with the revised version of the SeRA. First, a few respondents from non-Western migration backgrounds mentioned that they did not know whether items should be answered with the consequences of their health condition in mind, or with a more holistic focus on the whole person. In the first series of cognitive interviews with native Dutch persons, this concern did not occur and persons responded from the perspective of the whole person. Persons from different migration backgrounds possibly look different to health and disability (16). To clarify the perspective of the SeRA, we decided to adapt the instructions. Description of a clear instruction is an important part of comprehensibility of the content validity of a measure (12).

A few respondents showed to have more difficulty to answer some of the items compared to others. This difficulty may be linked to cognitive impairments or concentration issues. All interviews were conducted in person and so severe cognitive impairment likely would have been noticed, but we cannot be sure about this because we did not test for cognitive impairment prior to the interviews. A study among persons with mild to moderate cognitive impairments found that this population was able to respond consistently to questions on own preferences, their own involvement in decisions, and situations about daily living (26). Also, these difficulties were marked by persons in multiple diagnostic groups. Another explanation for slight variations in interpretation of the items may be found in the influence of personal factors. Personal factors are related to the understanding of functioning, disability, and health (27–29) and would always interfere to some extent in the answers provided. With this in mind it, can be argued that it is inevitable that there will be some slight deviations in the answering of the items, however not in understanding; therefore, this is no reason to make adaptations in the items.

## Strengths, limitations, and recommendations

A strength of this study is the methodological rigor, which was undertaken based on the COSMIN guidelines (12). Extensive investigation was done on the concept of self-regulation in the population and context of use (10). Furthermore, cognitive interviews were conducted in a multicultural post-rehabilitation sample, which strengthens the content validity of the SeRA.

Although the SeRA showed content validity according to the criteria for good content validity from the COSMIN, the results of this study need to be interpreted with caution due to the following limitations. First, respondents included in this study were former rehabilitation patients. Therefore, we cannot be sure the SeRA is also useful in a current rehabilitation population. It would be recommended to conduct additional psychometric testing of the SeRA among current rehabilitation patients, such as reliability and responsiveness. No rehabilitation professionals were included in the cognitive interviews, in contrast with what is recommended in the COSMIN criteria (15). However, a group of experts was involved during the phase of item generation. Future work to undertake translation and cultural adaption of the SeRA will be required to broaden its use internationally.

## Implications and relevance

From 2016 onward, multiple Dutch actors in the field of healthcare, such as the Minister of Healthcare and Federation of Medical Specialists, emphasized the need for structural evaluation of healthcare in the Netherlands (30). They recommend transparency of healthcare by quality registrations and information based on experienced daily functioning of patients. The position paper of the Netherlands Society of Rehabilitation Medicine describes to promote and retain the quality of life of their patients (3). Self-regulation directly influences a persons’ quality of life (31, 32). The SeRA was designed to measure conditional aspects of self-regulation and the application of self-regulation among rehabilitation patients. Desirably, the potential added value of this measure to set personal rehabilitation goals, identify patients with self-regulation problems at the start of rehabilitation, and measure outcomes of rehabilitation needs to be investigated in subsequent studies involving active rehabilitation patients.

## Conclusion

The SeRA showed content validity as a measure of self-regulation in a post-rehabilitation population, capturing all aspects conditional to, and to apply self-regulation. After revisions, deleting or adding items in the first series, comprehensibility, comprehensiveness, and relevance were confirmed in the second series of cognitive interviews with a former rehabilitation population with a non-Western migration background. This extensive investigation of the concept for a specific post-rehabilitation population distinguishes the SeRA from other measures.

## Data Availability

The raw data supporting the conclusions of this article will be available upon request.
